# Digitale Mediennutzung und psychische Gesundheit bei Adoleszenten – eine narrative Übersicht

**DOI:** 10.1007/s00103-024-03848-y

**Published:** 2024-03-01

**Authors:** Kerstin Paschke, Rainer Thomasius

**Affiliations:** Deutsches Zentrum für Suchtfragen des Kindes- und Jugendalters (DZSKJ), Universitätsklinikum Hamburg-Eppendorf, Martinistr. 52, 20246 Hamburg, Hamburg Deutschland

**Keywords:** Internet, Nutzungsstörungen, Mediensucht, Cybermobbing, Kinder und Jugendliche, Internet, Use disorder, Media addiction, Cyberbullying, Children and adolescents

## Abstract

Chancen und Gefahren digitaler Medien, vor allem im Hinblick auf Kinder und Jugendliche, sind gegenwärtig häufiger Gegenstand von familiären, schulischen und gesellschaftlichen Debatten.

Digitale Plattformen können die Bewältigung adoleszenter Entwicklungsaufgaben durch Spiele, sozialen Austausch, Kommunikation, Kontaktförderung, Lernen und Gesundheitsförderung unterstützen sowie zur Unterhaltung dienen. In Deutschland verfügen nahezu alle Jugendlichen über ein eigenes Smartphone. Während der COVID-19-Pandemie wurde eine Intensivierung der Nutzung digitaler Spiele, sozialer Medien und Streaming-Angebote durch Adoleszente beobachtet. Der Kontakt mit altersunzensierten Inhalten wie die Darstellung von Gewalt, extremen politischen Ansichten und Verschwörungstheorien, aber auch persönliche Angriffe durch Cybermobbing, ungefilterte Kontaktanbahnungen, inkl. Cybergrooming, dysfunktionale Rollenvorbilder und suchtfördernde Aspekte gehen mit Gefahren für die psychische Gesundheit einher.

Von Cybermobbing sind ca. 5 % der Kinder und Jugendlichen in Deutschland betroffen. Ein bidirektionaler Zusammenhang mit psychischer Gesundheit konnte gezeigt werden. Mit der Computerspielstörung ist die erste Digitale-Medien-Nutzungsstörung (DMNS) in die elfte Revision der Internationalen Klassifikation der Krankheiten (ICD-11) aufgenommen und damit als psychische Erkrankung international anerkannt worden. Adoleszente sind überproportional häufig betroffen und erfahren Beeinträchtigungen ihrer psychischen Entwicklung und ihres Funktionsniveaus.

Zur Förderung adoleszenter gesunder Mediennutzung stehen Angebote zur Verfügung, deren Ausbau, strukturierte Anwendung und Evaluierung erforderlich sind. Evidenzbasierte Präventions- und Behandlungsoptionen von DMNS fehlen derzeit weitgehend. Ihre Entwicklung, Überprüfung und Verbreitung sollten weiter gefördert werden.

## Abstract (en)

The opportunities and hazards of digital media, especially with regard to children and adolescents, are currently the subject of frequent family, school, and social debates.

Digital platforms can support the accomplishment of adolescent developmental tasks through gaming and social exchange as well as communication and contact promotion, learning, and health promotion, and are widely used for entertainment. In Germany, almost all adolescents own a smartphone. During the COVID-19 pandemic, an intensification of the use of digital games, social media, and streaming offers by adolescents was observed. Exposure to age-uncensored content such as the depiction of violence, extreme political views, and conspiracy theories, as well as personal attacks through cyberbullying, unfiltered initiation of contact including cybergrooming, dysfunctional role models, and addiction-promoting aspects are associated with mental health risks.

Cyberbullying affects about 5% of children and adolescents in Germany. A bidirectional relationship with mental health could be shown. The gaming disorder is the first digital media use disorder (DMUD) that has been included in the ICD-11 and thus internationally recognized as a mental illness. Adolescents are affected disproportionately often and experience impairments in their mental development and levels of functioning.

Services are available to promote adolescent media literacy, and their expansion, structured application, and evaluation are needed. Evidence-based prevention and treatment options for DMUD are currently largely lacking. Their development, review, and dissemination should be further supported.

## Hintergrund

Digitale Medien sind aus der heutigen Gesellschaft nicht mehr wegzudenken. Das Internet steht für universelle Konnektivität und gesellschaftlichen Wandel. In den letzten Jahrzehnten konnte eine rasante Entwicklung digitaler Technologien für den Privatgebrauch und deren flächendeckende Verfügbarmachung beobachtet werden. Die digitale Entertainment- und Medienindustrie gehört zu den am schnellsten wachsenden Geschäften weltweit. So wird ihr Umsatz im Jahr 2023 auf ca. 542,30 Mrd. € weltweit und davon 15 Mrd. € in Deutschland geschätzt [1].

### Verbreitung

Ein bedeutsamer Anstieg der Internetnutzenden auf aktuell ca. 5,3 Mrd. ist in allen Altersgruppen weltweit zu verzeichnen [1]. Kinder und Jugendliche des 21. Jahrhunderts werden als *Digital Natives *in die Welt der digitalen Medien hineingeboren und nehmen einen großen Anteil aller Nutzenden ein. Im Jahr 2022 gebrauchten in Deutschland unter den 10- bis 17-Jährigen 85 % digitale Spiele, 89 % soziale Medien und 83 % Video-Streaming-Dienste regelmäßig [2]. 96 % der 12- bis 19-Jährigen besitzen ein eigenes Smartphone und haben damit potenziell ständigen Zugang zu digitalen Inhalten. Im Durchschnitt sind sie täglich 3,4 h online [3]. Diese tägliche Nutzungszeit liegt über den z. B. von der Bundeszentrale für gesundheitliche Aufklärung (BZgA) empfohlenen maximalen 2,5 h [4]. Während der COVID-19-Pandemie war die Nutzungsintensität signifikant angestiegen [5].

### Chancen digitaler Mediennutzung

Digitale Medien bieten eine Vielzahl von Chancen und Potenzialen. Sie werden von Adoleszenten vorrangig zur Unterhaltung sowie zur Interaktion und Vernetzung mit Gleichaltrigen genutzt. Zudem helfen sie bei Selbstorganisation, Informationsgewinnung, Weiterbildung und Kommunikation im akademischen Kontext (z. B. durch individualisierte Lernprogramme und sog. Serious Games, Nutzung von Mail- und Online-Plattformen zum Austausch und zur Präsentation von schulischen Inhalten) sowie im Rahmen der Gesundheitsförderung (z. B. durch digitale Gesundheitsanwendungen). In Ergänzung zu realen Erfahrungen bieten digitale Medien zudem Plattformen zum zeitlich, örtlich und situativ flexiblen Bewältigen von adoleszenten Entwicklungsaufgaben wie der Entwicklung von Identität und Rolle in der Gesellschaft, von komplexen Handlungskompetenzen, Autonomie in Bezug auf erwachsene Bezugspersonen, Zukunftsperspektiven und eigenen Werte- und Moralüberzeugungen [6].

### Gefahren digitaler Mediennutzung

Neben positiven Aspekten digitaler Mediennutzung in der Adoleszenz geraten zunehmend mögliche Gefahren in das öffentliche Bewusstsein. Diese betreffen gesellschaftlich, politisch und juristisch relevante als auch direkte und indirekte gesundheitliche Aspekte im Rahmen problematischer Nutzungsmuster und Inhalte.

Bis zur Hälfte der 12- bis 19-Jährigen berichten davon, mit mindestens einem potenziell gefährdenden Inhalt in Berührung gekommen zu sein [3]. Dies beinhaltet den Kontakt mit und die Verbreitung von *Fake News*, beleidigenden Kommentaren, extremen politischen Ansichten und Propaganda, Verschwörungstheorien, Hassbotschaften (*Hate Speech*) und persönlichen Anfeindungen (inkl. Cybermobbing), Gewalt- und pornografische Darstellungen sowie Kontaktaufnahmen durch Fremde (inkl. *Cybergrooming*, d. h. die gezielte Anbahnung sexueller Kontakte mit Minderjährigen über das Internet).

Hinzu kommen potenzielle Beeinflussungen durch Werbeinhalte, Gefahren durch Betrügereien über Spam und Phishing sowie Schadprogramme, den Missbrauch persönlicher Daten, ungewollte kostenintensive Downloads und das Verlieren des Überblicks über finanzielle Ausgaben im Rahmen von *Click & Collect* oder *In-App*-Käufen bzw. *Freemium Games* (d. h. Spiele, die zunächst kostenfrei angeboten werden, bei denen weitere Nutzungsinhalte jedoch kostenpflichtig sind).

Außerdem wächst die Besorgnis im Zusammenhang mit teils riskantem Nachahmungsverhalten von Serieninhalten (z. B. reales Gewaltverhalten analog der Netflix-Serie *Squid Game*) und sog. *Challenges* (Erstellen und Teilen von Videos, in denen Aufgaben erfüllt werden, z. B. Selbststrangulation bis zum Bewusstseinsverlust im Rahmen der *Blackout*-Challenge auf TikTok).

Sorge besteht zudem über die Rolle von Medien bei der Förderung und Aufrechterhaltung selbstschädigender Verhaltensweisen und psychischer Probleme, wie im Rahmen von selbstverletzendem Verhalten, Suizidalität und Essstörungen (z. B. mit dem Hashtag #*thinspiration*, unter dem Untergewicht als erstrebenswerter Lifestyle und Anleitungen zum Abnehmen dargestellt werden sowie Vernetzung erfolgt).

### Besonderheiten der Adoleszenz

Die Adoleszenz umfasst die Altersspanne von 10 bis 19 Jahren und geht mit einer erhöhten Vulnerabilität für psychische Erkrankungen einher [7]. Adoleszente sollten daher nicht nur wegen ihrer hohen Affinität zu digitalen Medien besondere Berücksichtigung finden. Dem Bericht der Weltgesundheitsorganisation (WHO) zufolge sind 14 % der Adoleszenten weltweit von psychischen Erkrankungen betroffen [7]. Auf das gesamte Lebensalter gesehen, beginnen die meisten psychischen Erkrankungen im Mittel mit 14,5 Jahren [8]. Dies hat signifikanten Einfluss auf Wohlbefinden und Entwicklungsmöglichkeiten der Betroffenen sowie auf Gesundheitssystem und Volkswirtschaft.

Als ursächlich für die adoleszente Vulnerabilität werden neuronale Umbauprozesse diskutiert, die zu einem Reifungsungleichgewicht zwischen motivational-affektiven Hirnstrukturen und kognitiven Kontrollregionen zu Ungunsten der Letzteren führen [9]. Crone und Konijn beschreiben die damit im Zusammenhang stehende erhöhte emotionale Reaktivität, Belohnungssensitivität, vermehrte Inkaufnahme von Risiken, aber auch die erhöhte neuronale Suszeptibilität hinsichtlich sozialer Akzeptanz und Orientierung an *Peers* als bedeutsame Einflussfaktoren auf adoleszente Mediennutzung [10]. Die klinische Relevanz zeigt sich im systematischen Review von Schettler et al. [11], demzufolge eine erhöhte kognitiv-affektive neurobiologische Imbalance mit einer problematischen Nutzung von digitalen Spielen einhergeht.

### Mediennutzung und psychische Gesundheit

In der Literatur finden sich vermehrt Hinweise auf einen bidirektionalen Zusammenhang zwischen intensiver Mediennutzung und schlechterer mentaler Gesundheit bei Adoleszenten [12]. Gleichzeitig wird deutlich, dass im Zusammenhang mit psychischer Gesundheit nicht *die* Mediennutzung im Allgemeinen betrachtet werden kann, sondern Medieninhalte, Nutzungsmotive und Nutzungsmuster differenziert berücksichtigt werden müssen.

Diese Aspekte werden im vorliegenden narrativen Review näher beleuchtet. Hierbei erfolgt eine Fokussierung auf Nutzungsmotive, auf Cybermobbing als empirisch gut untersuchtes Beispiel gefährdender digitaler Interaktion und auf mit der Entstehung von Süchten assoziierte problematische Nutzungsmuster.

Für eine vereinfachte Lesbarkeit wird im Folgenden von *Medien* die Rede sein, auch wenn hierbei, im Gegensatz zu analogen, d. h. nicht interaktiv nutzbaren Medien wie das *klassische* Fernsehen, Zeitunglesen oder Radiohören, ausschließlich digitale Medien gemeint sind.

## Motive der Mediennutzung

Die *Uses-and-Gratifications*(U&G)-Theorie bietet einen häufig zitierten Rahmen, um zu verstehen, warum und wie Menschen Medien nutzen [13]. Adressiert werden soziale und psychologische Faktoren, die die Nutzenden bei ihrem Streben nach Zufriedenheit beeinflussen. Hierbei steht das Bestreben, persönliche Bedürfnisse und Wünsche zu erfüllen, im Vordergrund.

Nach McQuail beinhalten die Ziele der Mediennutzung Informationsgewinnung, Identitätsentwicklung, soziale Integration und Interaktion sowie Unterhaltung [14]. Die dahinterliegenden Bedürfnisse sind Katz et al. (1973) zufolge kognitiven, persönlichen, sozialen und affektiven Dimensionen zuzuordnen. Letztere beinhaltet neben der Induktion angenehmer Gefühle, die Vermeidung unangenehmer Gefühle und die Reduktion von Anspannung durch Eskapismus [15].

Stockdale et al. identifizierten das Motiv der sozialen Integration und die Vermeidung unangenehmer Gefühle (bzw. Bekämpfung von Langeweile) als Risikofaktor für problematische Nutzungsmuster sozialer Medien, wohingegen sich das Motiv der Informationsgewinnung als Schutzfaktor herausstellte [16].

Während der COVID-19-Pandemie zeigte Mediennutzung bei Adoleszenten protektive Einflüsse auf die mentale Gesundheit, wenn sie im Rahmen von 1:1-Kommunikation stattfand, Selbstoffenbarung bei gegenseitiger Online-Freundschaft beinhaltete sowie mit positiven und lustigen Erfahrungen zur Abmilderung von Einsamkeit und Stress einherging [17]. Auf der anderen Seite waren soziale Vergleichshandlungen, die Angst etwas zu verpassen (*Fear of Missing out* [FOMO]), aber auch der Kontakt mit negativen Inhalten mit reduzierter psychischer Gesundheit assoziiert.

Bei Adoleszenten mit vorbekannten psychischen Belastungen konnten im Rahmen eines systematischen Reviews folgende positive und negative Erfahrungen mit Medien und zugrunde liegende motivationale Faktoren identifiziert werden [18]: soziale Konnektivität und Unterstützung Gleichaltriger, Eskapismus und Ablenkung, soziale Validierung und sozialer Vergleich, Aufsuchen und Erzeugen von potenziell gefährdenden Inhalten, Cybermobbing sowie Schwierigkeiten in der Selbstregulation während der Mediennutzung. Eine reduzierte Selbstregulation ist hierbei mit problematischen Nutzungsmustern assoziiert [19].

## Cybermobbing

### Begrifflichkeiten und Kriterien

Forschung zu Cybermobbing ist unter einer Vielzahl von Begriffen zu finden, darunter *Cyber-Aggression, Internet Harassment, Online-Bullying* und *Electronic Bullying*. Im englischsprachigen Raum hat sich der Begriff des *Cyber Bullying* etabliert. Bislang existiert keine einheitliche Definition von Kriterien. Abgeleitet vom Mobbing in der realen Welt (*traditionelles* Mobbing) kann es als eine spezifische Form der Gewaltanwendung unter Nutzung von digitalen Endgeräten verstanden werden, die wiederholt mit der Absicht ausgeübt wird, andere zu schädigen, und bei den Adressaten Leid hervorruft [20].

Hierzu zählen der absichtliche Ausschluss von gemeinsamen Informationen, Online- oder persönlichen Interaktionen und die Verbreitung von Gerüchten (*relationales* Mobbing), aber auch Beleidigungen, Belästigungen, Verleumden, Schikanieren oder Drohungen (*verbales* Mobbing; [21]). Im Gegensatz zu traditionellem Mobbing können Botschaften weitgehend zeit-, situations- und ortsunabhängig ausgesandt und empfangen sowie ohne persönlichen Kontakt breit disseminiert werden. Durch die Möglichkeit, anonym zu agieren, ist ein Machtungleichgewicht nicht zwingend erforderlich. Cybermobbing und traditionelles Mobbing geschehen häufig parallel oder gehen ineinander über [22].

### Prävalenzen

Durch differierende Definitionen, Operationalisierungen und Erhebungsmethoden variieren Prävalenzraten zwischen Studien oft erheblich [23]. International werden Erfahrungen mit Mobbing im Internet bei 13,9–57,5 % bzw. das Ausübung von Cybermobbing bei 6–46,3 % der Kinder und Jugendlichen berichtet [20].

Den Ergebnissen der deutschen *Health-Behaviour-in-School-aged-Children*(HBSC)-Studie von 2017/2018 (*N* = 4347 Lernende; 53,0 % weiblich) zufolge, berichten 3,9 % der Schülerinnen und Schüler von digitalen Mobbingkontakten (1,3 % ausübend; 2,0 % erfahrend; 0,6 % beide Rollen; [24]). Die Prävalenz von traditionellem Mobbing liegt mit 13,3 % (3,9 % ausübend; 8,3 % erfahrend; 1,1 % beide Rollen) deutlich höher.

Die Gegenüberstellung der Aktualprävalenzen des Cybermobbings in 19 Mitgliedsstaaten der Europäischen Union weist für Deutschland mit 5 % ein vergleichsweise niedriges Niveau auf [25]. Die höchsten Prävalenzen dafür, Opfer von Cybermobbing zu werden, finden sich bei Adoleszenten in Polen (31,5 %) und Tschechien (18,6 %), die niedrigsten in Italien und Portugal (2,8 %). Daten für die Ausführung des Cybermobbings existieren aus 4 Ländern: Polen (30,6 %), Tschechien (10,5 %), Slowenien (3,0 %) und Finnland (9 %). Daten für das gleichzeitige Auftreten von Cybermobbing in der Täter- und Opferrolle liegen aus Finnland vor (4 %).

Am stärksten stieg die Prävalenz für das Erfahren von Cybermobbing zwischen 2010 und 2015 in Spanien (von 5 % auf 12 %) sowie zwischen 2010 und 2018 in Tschechien (von 9,4 % auf 18,6 %) und in Polen (von 7 % auf 31,5 %). Während der COVID-19-Pandemie wurden international bei Kindern und Jugendlichen sowohl zunehmende (v. a. in asiatischen Ländern und Australien) als auch gleichbleibende und abnehmende Trends bei Cybermobbingerfahrungen (v. a. in westlichen Ländern) beobachtet [26]. Einschränkend muss zu den Ergebnissen dieses systematischen Reviews gesagt werden, dass die meisten von den 16 inkludierten Studien querschnittlich mit retrospektiver Befragung angelegt waren.

### Ätiologie

In Bezug auf die Ätiologie von Cybermobbing im Kindes- und Jugendalter werden persönliche, familiäre und soziale sowie situative Faktoren diskutiert [20]. Als Risikofaktoren für das Erleben von Cybermobbing wurden hierbei beschrieben: weibliches Geschlecht, hohe Nutzungszeiten digitaler Medien, psychische Probleme, Einsamkeit, geringer Selbstwert, elterliche Vernachlässigung und vermindertes und inkonsistentes elterliches Monitoring, gestörte familiäre Kommunikation sowie Aufwachsen in Großstädten.

Hinsichtlich der Ausübung von Cybermobbing wird angenommen, dass insbesondere Jugendliche über 15 Jahre ein erhöhtes Risiko aufweisen, in die Täterposition zu gelangen, vor allem bei hoher Ausprägung von Impulsivität, Verhaltensproblemen, Hyperaktivität, reduziertem prosozialen Verhalten, Substanzmissbrauch, überkontrollierenden und autoritären Erziehungsstilen in der Herkunftsfamilie sowie schulischen Interaktionsproblemen und eigenen Mobbingerfahrungen.

Demgegenüber konnten hohe sozioemotionale Fertigkeiten sowie Fähigkeit zur Selbstkontrolle, Empathie, positive Eltern-Kind-Beziehungen und Peer-Interaktionen sowie ein positives Schulklima als Schutzfaktoren für das Cybermobbing in Kindheit und Jugend identifiziert werden [20, 25, 27].

### Cybermobbing und psychische Gesundheit

Cybermobbing geht mit internalisierenden und externalisierenden Problemen einher [20]. Adoleszente Betroffene von Cybermobbing berichten vermehrt Ängste, psychosomatische Beschwerden, Schlafprobleme, selbstschädigende Verhaltensweisen und Suizidgedanken [28]. Längsschnittliche Studien konnten einen Zusammenhang zwischen dem Erleben von Cybermobbing und späteren depressiven Symptomen zeigen [21]. Gleichzeitig erleben Kinder und Jugendliche mit psychischen Problemen häufiger Cybermobbing [22]. Ausführende von Cybermobbing zeigen häufiger Substanzmissbrauch und Impulskontrollstörungen [27].

## Problematische Mediennutzung

### Begrifflichkeiten und Kriterien

In den letzten 3 Jahrzehnten ist eine Vielzahl von Begriffen in der Literatur zur Beschreibung von Nutzungsmustern digitaler Medien mit Suchtcharakteristika genannt worden, die die Vergleichbarkeit von Befunden stark erschwerte. Diese beinhalten u. a. Internetsucht, pathologischen Internetgebrauch, exzessive oder zwanghafte Internetnutzung, aber auch Handysucht.

Zunehmend haben sich eine Fokussierung auf das zugrunde liegende Verhalten anstatt auf das Endgerät sowie eine Differenzierung von problematischen Anwendungen durchgesetzt, z. B. in Bezug auf die Nutzung digitaler Spiele sowie sozialer Netzwerke [29]. In Anlehnung an die Terminologie für Substanzgebrauchsstörungen der internationalen statistischen Klassifikation der Krankheiten und verwandter Gesundheitsprobleme (ICD-)10 bzw. ICD-11 und unter Berücksichtigung von digitalen Online- und Offline-Medien wurden die Oberbegriffe der medienbezogenen Störungen oder Digitale-Medien-Nutzungsstörungen (DMNS) vorgeschlagen [30]. Pathologische und riskante Nutzungsmuster werden als problematische Verhaltensweisen zusammengefasst.

Als erste rein mit digitalen Medien assoziierte Verhaltenssucht wurde die problematische Nutzung digitaler Spiele in die aktuelle (11.) Version der ICD (ICD-11) als Computerspielstörung (*Gaming Disorder *[GD] 6C51) aufgenommen [31]. Ihre Kriterien sind in Tab. 1 aufgeführt.

**Table 1:** 

Verlust der Kontrolle über die zeitliche und situative Nutzung digitaler Spiele
Verstärkte Priorisierung des digitalen Spielens gegenüber alternativen Aktivitäten
Fortgesetztes oder verstärktes Spielen trotz negativer Konsequenzen
Das Auftreten erheblicher Beeinträchtigungen in wichtigen Funktionsbereichen
Verhalten bestand in der Regel in den letzten 12 Monaten
Alle Kriterien müssen für eine GD erfüllt sein

*ICD-11* 11. Version der internationalen statistischen Klassifikation der Krankheiten und verwandter Gesundheitsprobleme

Weitere Störungen im Zusammenhang mit der Nutzung digitaler Medien können unter „Andere spezifische Störungen aufgrund von Suchtverhalten (GC5Y)“ in der ICD-11 klassifiziert werden [29]. Ausgehend von den Statistiken über die Nutzungshäufigkeit bei Adoleszenten erscheinen in dieser Altersgruppe neben der GD vor allem die problematische Nutzung von sozialen Medien und Video-Streaming-Diensten bedeutsam. Ihre Relevanz ist Gegenstand intensiver wissenschaftlicher Debatten. Ein (noch) unvollständiges Bild einer Sucht kann als Gesundheitsproblem unter den Begrifflichkeiten „Gefährliches Spielen (Hazardous Gaming [HG] QE22)“ oder „Probleme mit anderen spezifizierten gesundheitsbezogenen Verhaltensweisen (QE2Y)“ eingeordnet werden.

Vergleichbare, an deutschen adoleszenten Repräsentativstichproben validierte Fragebögen zur Erfassung der ICD-11-Kriterien in Bezug auf die 3 genannten DMNS bei Adoleszenten sind für ein Selbst- und für ein Elternurteil verfügbar (Tab. 2).

**Table 2:** 

DMNS	Fragebogen	Adressat
Computerspielstörung	Gaming Disorder Scale for Adolescents, GADIS‑A [32]	Adoleszente ab 10 Jahren (Selbsturteil)
Computerspielstörung	Gaming Disorder Scale for Parents, GADIS‑P [33]	Eltern von Adoleszenten (Fremdurteil)
Soziale-Netzwerke-Nutzungsstörung	Social Media Use Disorder Scale for Adolescents, SOMEDIS‑A [34]	Adoleszente ab 10 Jahren (Selbsturteil)
Soziale-Netzwerke-Nutzungsstörung	Social Media Use Disorder Scale for Parents, SOMEDIS‑P [35]	Eltern von Adoleszenten (Fremdurteil)
Video-Streaming-Störung	Streaming Disorder Scale for Adolescents, STREDIS‑A [36]	Adoleszente ab 10 Jahren (Selbsturteil)
Video-Streaming-Störung	Streaming Disorder Scale for Parents, STREDIS‑P [37]	Eltern von Adoleszenten (Fremdurteil)

*ICD-11* 11. Version der internationalen statistischen Klassifikation der Krankheiten und verwandter Gesundheitsprobleme

### Prävalenzen

Bei einigen Nutzenden kann sich ein regelmäßiger Freizeitkonsum über einen intensiven Konsum zu problematischen Verhaltensmustern entwickeln [19]. Metaanalysen schätzen die weltweite Prävalenz für die problematische Nutzung digitaler Spiele auf 3,3 % [38] und sozialer Medien auf 5 % [39], wobei Adoleszente am häufigsten betroffen sind.

Weitaus weniger Daten gibt es zu problematischem Video-Streaming. Eine erste repräsentative Studie schätzt die Prävalenz von Adoleszenten in Deutschland, die regelmäßig Video-Streaming-Angebote nutzen, auf 4,7 % [36]. Während der COVID-19-Pandemie haben problematische Nutzungsmuster bei Kindern und Jugendlichen aufgrund von Kontaktbeschränkungen, Quarantänen sowie der Schließung von Schulen und Freizeiteinrichtungen zugenommen [5].

### Ätiologie

Die Ätiologie von DMNS ist komplex und kann z. B. durch das *Interaction-of-Person-Affect-Cognition-Execution*(I-PACE)-Modell mit dem Fokus auf die Wechselwirkung intra- und interindividueller Prozesse beschrieben werden [40].

Ein weiteres etabliertes Modell zum Verständnis der Entstehung und Aufrechterhaltung von Süchten im Allgemeinen ist das Trias-Modell der Sucht von Kielholz und Ladewig [41]. Dieses biopsychosoziale Modell beschreibt eine Interaktion von persönlichen, sozialen und medienimmanenten Faktoren. Es bezieht Mechanismen des Lernens, der Stressbewältigung sowie der Informationsverarbeitung mit ein. In Bezug auf DMNS umfassen persönliche Faktoren Persönlichkeitsmerkmale wie Neurotizismus, *Sensation Seeking* und verminderte Gewissenhaftigkeit [42, 43], Probleme mit der Emotionsregulation, einschließlich Prokrastination, Vermeidung und verminderter Wutkontrolle [44, 45], sowie mangelhafte soziale Fähigkeiten [46].

Darüber hinaus spielen elterliche und familiäre Faktoren wie das elterliche Rollenmodell, das Erziehungsverhalten, die intrafamiliäre Kommunikation, das familiäre Funktionsniveau und die Beziehungen, aber auch Peer- und gesellschaftliche Einflüsse eine wesentliche Rolle bei der Entwicklung [47].

Zu den medienimmanenten Faktoren gehören Mechanismen der Leistungs‑, Emotions- und Überzeugungsgestaltung, wie der Einsatz von (intermittierenden) Verstärkungslernstrategien zur Verhaltenskonsolidierung und zur Vermeidung von Verhaltenslöschungen (sog. *Dark Patterns;* [48]).

### Problematische Mediennutzung und psychische Gesundheit

Das Vollbild einer DMNS geht mit einer signifikanten Beeinträchtigung der psychischen Gesundheit einher und verläuft ohne adäquate Behandlung nicht selten chronisch. In der Regel finden sich hier psychische Erkrankungen wie Depression, Angst- und Zwangs‑, Somatisierungs- sowie Schlafstörungen komorbid, wobei die DMNS zeitlich sowohl vor- als auch nachgeordnet sein können.

Hinzu werden DMNS gehäuft bei Adoleszenten mit einfacher Aktivitäts- und Aufmerksamkeitsstörung, Autismusspektrumstörungen und Störungen des Sozialverhaltens beobachtet [49]. Riskante Nutzungsmuster können häufiger spontan remittieren [30]. Doch zeigen auch Adoleszente mit riskanten Nutzungsmustern bereits vermehrt psychische Belastungen, wobei die Kausalität bidirektional angenommen werden muss [32, 34, 36].

## Förderung einer gesunden Mediennutzung

### Medienkompetenz und Prävention

In den Rahmenlehrplänen der Bundesländer wurde die integrale und fachübergreifende Bedeutung digitaler Medien im Schulunterricht wiederholt festgeschrieben. Diese Vorgaben sind in den länderspezifischen Medienkompetenzrahmen für alle Klassenstufen und Unterrichtsfächer definiert. Sie bilden damit verbindliche Grundlagen für die Entwicklung eines sicheren und verantwortungsvollen Umgangs von Kindern mit Medien über die Vermittlung von mediennutzungsbezogenem Grundwissen.

Online existieren zahlreiche professionelle Plattformen zur Förderung einer gesunden Medienentwicklung und Sensibilisierung hinsichtlich Cybermobbings und problematischer Nutzungsmuster bei Kindern und Jugendlichen (Tab. 3). Grund- und weiterführende Schulen stellen zudem einen geeigneten Ort für die Implementierung effektiver Präventionsstrategien dar, um Kinder über Gefahren zu informieren, sie zu einem sicheren Gebrauch digitaler Medien anzuregen und negative psychosoziale Konsequenzen im Zusammenhang mit Cybermobbing zu vermeiden [22, 28].

**Table 3:** 

Name	Herausgebende/Förderer	Inhalte/Adressaten	Webseite
Schau Hin	BZgA	Medienkompetenz für Eltern	https://www.schau-hin.info
Klicksafe	Gefördert durch das Digital Europe Programm DIGITAL der Europäischen Union, koordiniert von der Medienanstalt Rheinland-Pfalz, umgesetzt gemeinsam mit der Landesanstalt für Medien NRW	Förderung von Online-Kompetenz, Schulungen für Kinder, Jugendliche, Eltern, Lehr- und Fachkräfte	https://www.klicksafe.de
Gutes Aufwachsen mit Medien	Gefördert vom BMFSFJ	Medienerziehung in der Familie	https://www.gutes-aufwachsen-mit-medien.de
Ins Netz gehen	BZgA	Informationen für Kinder und Jugendliche zu gesunder Mediennutzung, Selbsttest, Online-Beratung	https://www.ins-netz-gehen.de
Internet-abc	Medienkompetenz-Initiative der deutschen Landesmedienanstalten	Informationen für Kinder, Eltern, Lehrkräfte, Unterrichtseinheiten, Lernmodule, Tipps zu Medienkompetenz	https://www.internet-abc.de/
Jugendschutz.net	Gemeinsames Kompetenzzentrum von Bund und Ländern für den Schutz von Kindern und Jugendlichen im Internet	Prüfung von Angeboten im Netz auf Verstöße gegen den Jugendschutz, Informationen, Aufklärungsmaterialien für Kinder und Jugendliche	https://www.jugendschutz.net
Spieleratgeber-NRW	Fachstelle für Jugendmedienkultur NRW, gefördert vom Ministerium für Kinder, Jugend, Familie, Gleichstellung, Flucht und Integration des Landes Nordrhein-Westfalen	Pädagogische Beurteilung von digitalen Spielen	https://spieleratgeber-nrw.de/
Cybermobbing/Medienpädagogische Beratungsstelle	Landesmedienzentrum Baden-Württemberg	Informationen und Unterrichtsmedien zu Cybermobbing	https://www.lmz-bw.de/medienbildung/themen-von-a-bis-f/cybermobbing/
Mediensuchthilfe	DZSKJ am Universitätsklinikum Hamburg-Eppendorf gefördert über das BMFSFJ und die DAK-Gesundheit	Broschüren und Online-Informationen, interaktiver Selbsttest zu problematischen Nutzungsmustern	https://www.mediensuchthilfe.info

*BMFSFJ* Bundesministerium für Familie, Senioren, Frauen und Jugend, *BZgA* Bundeszentrale für gesundheitliche Aufklärung, *DZSKJ* Deutsches Zentrum für Suchtfragen des Kindes- und Jugendalters, *NRW* Nordrhein-Westfalen

Das Programm „Medienhelden“ zur Verminderung von Cybermobbing und Verbesserung von Medienkompetenz richtet sich an Siebt- bis Zehntklässler und Lehrkräfte [50]. In seiner 10-Wochen-Lang- bzw. 1‑Tages-Kurzversion dient es der Förderung prosozialen medienbezogenen Verhaltens. Im Rahmen der Effektivitätsprüfung zeigte die Wartekontrollgruppe gegenüber der Langinterventionsgruppe nach Abschluss des Programms signifikant mehr (Bereitschaft zu) Cybermobbing.

Mit PROTECT (Professioneller Umgang mit technischen Medien) steht ein kognitiv-verhaltenstherapeutisches (KVT) und modellbasiertes Programm zur Prävention von DMNS bei Schülerinnen und Schülern ab 12 Jahren mit riskanten Nutzungsmustern zur Verfügung [51]. In einer clusterrandomisierten klinischen Studie konnte die Effektivität des Programms hinsichtlich einer Symptomreduktion gezeigt werden.

Im Rahmen indizierter Prävention kann das kognitiv-behaviorale Gruppenprogramm „Lebenslust statt Onlineflucht“ eingesetzt werden [52]. Es richtet sich an 14- bis 19-jährige Jugendliche mit problematischer Nutzung digitaler Spiele und verwendet motivationale, psychoedukative, verhaltensmodifizierende und stabilisierende Elemente. Die Ergebnisse der Pilotstudie sind hinsichtlich der Abnahme von problematischen Nutzungsmustern und Nutzungszeiten vielversprechend.

### Behandlung

Derzeit fehlt es weitgehend an modellbasierten Behandlungsprogrammen von DMNS im klinischen Kontext, insbesondere für die gesamte Altersspanne der Adoleszenz. Darüber hinaus weisen die verfügbaren Interventionsstudien große Qualitätsunterschiede auf, was ihre Vergleichbarkeit stark einschränkt [53].

Die besten Effekte auf die Symptomreduzierung wurden für Programme der KVT nachgewiesen [54]. Trotz der Forderung von klinisch Tätigen und Forschenden, die Familie in die Behandlung betroffener Jugendlicher einzubeziehen [55], ist dies nur in wenigen Programmen und teilweise in sehr begrenztem Umfang realisiert [56].

Das neue Programm Res@t (Ressourcenstärkendes Adoleszenten- und Eltern-Training bei Medienbezogenen Störungen) versucht diese Lücke zu schließen [57]. Hierbei handelt es sich um ein manualisiertes KVT-Programm, das ursprünglich für 10- bis 19-Jährige mit problematischer Nutzung digitaler Spiele (Res@t-A) und deren Eltern (Res@t-P) in einem Gruppensetting entwickelt wurde. Pilotstudiendaten von Res@t‑P im Rahmen eines Prä-post-follow-up-Designs zeigten vielversprechende Effekte: eine Abnahme des elterlichen Stressempfindens und eine Zunahme der familiären Funktionsfähigkeit sowie Symptomreduktion bei betroffenen Adoleszenten [56].

Res@t wurde mithilfe einer digitalen Anwendung um die Adressierung der problematischen Nutzung von sozialen Medien und Video-Streaming-Diensten für eine individuelle Nutzung erweitert [58]. Die automatisierte App soll eine Unterstützung in Ergänzung zu lokal verfügbaren Behandlungsoptionen bzw. den Einsatz im Rahmen einer *Blended Therapy* ermöglichen. Für die Evaluation der Wirksamkeit haben im Februar 2024 die Erhebungen im Rahmen einer multizentrischen randomisiert kontrollierten Studie in einem deutschlandweiten Konsortium aus kinder- und jugendpsychiatrischen Kliniken und Praxen (https://www.uke.de/projekte/resat). Die Ergebnisse der durch den Innovationsfond des Gemeinsamen Bundesausschusses (G-BA) geförderten Studie werden 2025 präsentiert werden.

## Fazit

Digitale Medien bieten bedeutende Chancen im Rahmen intra- und interindividueller sowie gesellschaftlicher Entwicklungen. Nahezu alle Jugendlichen besitzen ein eigenes Smartphone und haben damit potenziell zeitlich und örtlich unbeschränkten Zugriff auf digitale Inhalte. Aufgrund realer Gefahren durch die Verbreitung ungefilterter Inhalte und suchtfördernde Aspekte steigt die Sorge um die adoleszente psychische Gesundheit.

Das Forschungsfeld zum Zusammenhang digitaler Mediennutzung und psychischer Gesundheit ist äußerst heterogen. Eine Vielfalt von Begrifflichkeiten und differierenden Konzepten in Kombination mit einer großen Bandbreite an Forschungsqualität schränkt die Vergleichbarkeit von Studien und die Generalisierbarkeit der Befunde stark ein.

Doch insgesamt wird deutlich, dass nicht das digitale Endgerät oder die reine Zeit, die damit verbracht wird, ausschlaggebend im Hinblick auf gesundheitsbezogene Aspekte ist. Vielmehr muss die Qualität der Nutzung hinsichtlich der Inhalte und der Verhaltensmuster differenziert betrachtet werden.

So existiert eine große wissenschaftliche Evidenz für den Zusammenhang zwischen psychischen Problemen und den Kontakt mit Cybermobbing sowie mit problematischen Nutzungsmustern, die sich durch Kontrollverlust und Priorisierung zugunsten der Mediennutzung sowie die Inkaufnahme negativer Folgen und Beeinträchtigungen des Funktionsniveaus auszeichnen. Dabei konnten die Medienerfahrungen sowohl als Ursache als auch als Folge psychischer Probleme identifiziert werden.

Die problematische Nutzung digitaler Spiele ist als Computerspielstörung als psychische Erkrankung international anerkannt. Sie geht in der Regel mit psychischen Komorbiditäten und schwerwiegenden negativen persönlichen, familiären, sozialen, akademischen und beruflichen Folgen einher [59].

Inwieweit andere DMNS, z. B. im Zusammenhang mit sozialen Medien oder Streaming-Diensten, selbst Krankheitswert haben, ist derzeit Gegenstand intensiver Debatten. Unabhängig vom Ausgang dieser Debatten ist klar, dass schon jetzt die Bedarfe an wirksamen Präventionskonzepten und Interventionsprogrammen die Verfügbarkeit dieser deutlich überschreiten.

Die Vermittlung eines gesunden Umgangs von Kindern und Jugendlichen mit digitalen Medien in einer Zeit, in der nicht mehr auf digitale Medien verzichtet werden kann, ist zentrales Element, um die Chance auf eine gesunde Entwicklung zu gewährleisten. Hierbei ist die Unterstützung der Kinder und Jugendlichen beim Erwerb der Medienkompetenz durch Eltern und Schule essenziell. Gleichzeitig müssen flächendeckende, evidenzbasierte Präventions- und Behandlungsangebote geschaffen werden. Politik und Gesellschaft sind gefragt einen optimalen Rahmen zu bilden, damit diese besondere Herausforderung des 21. Jahrhunderts erfolgreich bewältigt werden kann.
